# Clinical Characteristics, Etiologies, and Outcomes of Adults Presenting With Altered Mental Status to the Emergency Department: A Retrospective Cohort Study

**DOI:** 10.7759/cureus.117634

**Published:** 2026-10-08

**Authors:** Bilawal Ali, Amber Naz, Hammadia Khalid, Ahmed Khalil, Momina Naz, Afshal Ali, Aisha Butt

**Affiliations:** 1 Internal Medicine, Allama Iqbal Teaching Hospital, Dera Ghazi Khan, PAK; 2 Internal Medicine, Bahria International Hospital, Lahore, PAK; 3 Emergency Medicine, M Islam Teaching Hospital, Gujranwala, PAK; 4 Biochemistry, Peshawar Medical College, Peshawar, PAK; 5 Neurosurgery, Rehman Medical Institute, Peshawar, PAK; 6 Internal Medicine, Qadir Ali Hospital, Layyah, PAK; 7 General Surgery, Services Hospital, Lahore, PAK

**Keywords:** altered mental status, coma, delirium, disorientation, emergency department, glasgow coma scale score, lethargy

## Abstract

Background

Altered mental status (AMS) is a common emergency presentation with diverse neurological, infectious, metabolic, toxicological, and systemic causes.

Objective

The objective of this study was to describe the clinical characteristics, underlying etiologies, management patterns, and clinical outcomes of adults presenting to the emergency department with AMS, and to identify independent clinical factors associated with in-hospital mortality.

Methods

This retrospective cohort study was conducted at Allama Iqbal Teaching Hospital, Dera Ghazi Khan, Pakistan, from April 2024 to April 2026 and included 210 adult patients presenting with AMS. Demographic and baseline clinical characteristics, including age, gender, body mass index, smoking status, presenting symptoms, duration of AMS, known comorbidities, medication history, substance use, and relevant previous neurological or systemic illnesses, were recorded.

Results

Of 210 patients, 174 (82.9%) survived, and 36 (17.1%) died. The mean age was 61.74±16.64 years, and 118 (56.2%) were male. Neurological causes were most common (29.0%), followed by infectious (21.4%), metabolic/endocrine (16.2%), and toxicological causes (11.9%). Non-survivors were older than survivors (72.10±13.80 vs. 59.60±16.40 years, p<0.001) and had lower Glasgow Coma Scale (GCS) scores (6.90±3.20 vs. 11.60±3.10, p<0.001), lower oxygen saturation (89.10±8.00% vs. 94.60±4.40%, p<0.001), and higher serum lactate levels (4.80±2.70 vs. 2.30±1.60 mmol/L, p<0.001). Intensive care unit (ICU) admission was required in 61 (29.0%) patients, while 42 (20.0%) required mechanical ventilation.

Conclusion

Altered mental status was associated with considerable ICU utilization and in-hospital mortality. Neurological and infectious causes predominated, while severe impairment of consciousness, elevated lactate, hypoxemia, hypotension, infectious etiology, and advanced age were independently associated with increased odds of mortality.

## Introduction

Altered mental status (AMS) is a common and clinically important presentation in the emergency department (ED) and encompasses disturbances in cognition, attention, awareness, behavior, and level of consciousness [1]. The clinical spectrum ranges from confusion, disorientation, agitation, and delirium to lethargy, stupor, and coma, depending on the severity and underlying cause [2]. AMS is a clinical syndrome rather than a specific disease entity and may result from a broad range of neurological, metabolic, infectious, toxicological, cardiovascular, psychiatric, and systemic conditions [3]. Its nonspecific presentation can make early diagnosis challenging, particularly in the emergency setting, where prompt recognition of potentially life-threatening and reversible causes is essential [4]. The underlying etiological spectrum of AMS varies according to age, comorbidities, medication exposure, substance use, environmental factors, and the characteristics of the population being evaluated [5].

Neurological disorders such as acute ischemic or hemorrhagic stroke, seizures and postictal states, traumatic brain injury, intracranial tumors, and central nervous system infections are important causes of AMS [6]. Systemic and metabolic disturbances, including hypoglycemia, electrolyte abnormalities, hypoxemia, hepatic encephalopathy, uremia, and acid-base disorders, may also cause impaired consciousness or cognitive dysfunction [7]. In older and medically vulnerable adults, serious infections such as pneumonia, urinary tract infections, and sepsis may initially manifest primarily as confusion or delirium rather than with typical symptoms of infection [8]. Toxicological and medication-related causes, including drug toxicity, adverse drug effects, alcohol intoxication, and intentional or unintentional poisoning, are also important considerations in patients presenting with AMS [9].

Older adults are particularly vulnerable to AMS because acute delirium may be the first or only manifestation of a serious underlying illness [10]. Advanced age, polypharmacy, pre-existing cognitive impairment, dehydration, and multiple chronic diseases may further increase susceptibility and adversely affect clinical outcomes [11]. In younger adults, seizures, trauma, substance-related disorders, toxic exposures, and acute neurological conditions may represent a greater proportion of presentations [12]. The evaluation of AMS requires a systematic approach beginning with stabilization of the airway, breathing, and circulation, followed by rapid identification and treatment of potentially reversible causes [13]. Depending on the clinical presentation, assessment may include bedside blood glucose measurement, neurological examination, Glasgow Coma Scale (GCS) assessment, laboratory investigations, electrocardiography, toxicological testing, and neuroimaging [14]. Because patients with AMS may be unable to provide a reliable history, information obtained from family members, caregivers, emergency medical personnel, and previous medical records may be essential for establishing the onset, clinical course, and likely underlying cause [15].

Given the broad etiological spectrum and potentially serious outcomes associated with AMS, characterization of its clinical presentation, underlying causes, management requirements, and prognostic factors is important for optimizing emergency assessment and resource utilization. However, the clinical profile and outcomes of patients presenting with AMS may vary across healthcare settings and patient populations. Therefore, the present study aimed to describe the clinical characteristics, underlying etiologies, management patterns, and clinical outcomes of adults presenting to the ED with AMS, and to identify independent clinical factors associated with in-hospital mortality.

Objective

The objective of this study was to describe the clinical characteristics, underlying etiologies, management patterns, and clinical outcomes of adults presenting to the ED with AMS, and to identify independent clinical factors associated with in-hospital mortality.

## Materials and methods

Study design and setting

This retrospective cohort study was conducted at Allama Iqbal Teaching Hospital, Dera Ghazi Khan, Pakistan, from April 2024 to April 2026. The study included 210 adult patients presenting to the ED with AMS. The underlying cause of AMS was determined from the clinical history, examination findings, laboratory and imaging investigations, and final hospital diagnosis documented in the medical record. As this was a retrospective review of existing medical records and involved no direct participant intervention, the requirement for individual informed consent was waived. Patient confidentiality was maintained throughout the study, and no personally identifiable information was included in the study database or manuscript.

Inclusion and exclusion criteria

Patients aged ≥18 years with an acute or subacute alteration in cognition, awareness, behavior, attention, and/or level of consciousness were eligible for inclusion. Altered mental status was defined as a documented acute change from the patient's usual mental state, ranging from confusion, disorientation, agitation, or delirium to lethargy, stupor, or coma. Patients were included if they had a documented initial ED assessment, sufficient clinical and laboratory information for evaluation of the presenting condition, and a documented final diagnosis and clinical outcome. Patients aged <18 years, those with chronic cognitive impairment or established dementia without an acute change in mental status, patients pronounced dead before arrival or at the scene, those transferred to the study hospital after receiving definitive treatment elsewhere, and patients with incomplete medical records or unavailable clinical outcomes were excluded.

Data collection

Eligible patients were identified from the electronic and/or medical records of the ED using consecutive sampling. Demographic and baseline clinical characteristics, including age, sex, body mass index (BMI), smoking history, presenting symptoms, duration of AMS, known comorbidities, medication history, substance use, and relevant previous neurological or systemic illnesses, were recorded.

Initial clinical parameters included systolic and diastolic blood pressure, heart rate, respiratory rate, peripheral oxygen saturation (SpO₂), body temperature, random blood glucose level, and GCS score at ED presentation. Relevant examination findings, including focal neurological deficits, seizures, fever, neck stiffness, evidence of trauma, agitation, and other signs of systemic illness, were also recorded.

Laboratory investigations, including complete blood count, serum electrolytes, renal function tests, liver function tests, blood glucose, arterial blood gas analysis, and serum lactate, were recorded from the initial ED assessment. Inflammatory markers and toxicological screening were recorded when available or clinically indicated. Arterial blood gas analysis, including arterial pH and serum lactate measurements, was available for all included patients and was therefore analyzed with complete-case denominators in the study population. Neuroimaging findings from computed tomography (CT) or magnetic resonance imaging (MRI) of the brain were documented when performed and were used to support etiological classification where available.

The underlying etiology of AMS was classified into eight predefined categories: neurological, infectious, metabolic/endocrine, toxicological/drug-related, traumatic, cardiopulmonary, psychiatric, and multifactorial. Etiological classification was based on the final clinical diagnosis established during hospitalization by the treating clinical team and supported by the available history, physical examination, laboratory investigations, neuroimaging, microbiological testing, and other relevant diagnostic findings. Neurological causes included acute cerebrovascular events, seizures/postictal states, and other acute neurological disorders; infectious causes included sepsis and clinically diagnosed infections involving the central nervous system or other organ systems; metabolic/endocrine causes included hypoglycemia, electrolyte disturbances, hepatic encephalopathy, and uremic encephalopathy; toxicological/drug-related causes included medication toxicity, alcohol or substance intoxication, and poisoning. Traumatic, cardiopulmonary, and psychiatric causes were classified according to the documented primary clinical diagnosis. Cases with two or more clinically significant conditions considered to contribute to AMS were classified as multifactorial.

Management variables included airway intervention, supplemental oxygen therapy, intravenous fluid administration, antimicrobial therapy, anticonvulsant treatment, specific antidote administration, vasopressor support, endotracheal intubation and mechanical ventilation, intensive care unit (ICU) admission, and specialist consultation.

Clinical outcomes included disposition from the ED, hospital admission, ICU admission, duration of hospital stay, neurological recovery, in-hospital complications, requirement for mechanical ventilation, and in-hospital mortality. Neurological recovery was defined as documented improvement in mental status to the patient's premorbid or near-premorbid cognitive and level-of-consciousness status by the time of hospital discharge, based on the treating team's clinical documentation and, where available, comparison with the patient's documented baseline neurological status.

Statistical analysis

Data were analyzed using IBM SPSS Statistics version 26.0 (IBM Corp., Armonk, NY, USA). Continuous variables were assessed for distribution and are presented as mean ± standard deviation (SD) for normally distributed variables. Categorical variables were presented as frequencies and percentages.

For comparisons between survivors and non-survivors, all continuous variables included in the comparative analysis were normally distributed and were therefore analyzed using the independent-samples t-test. Categorical variables were compared using the chi-square test or Fisher's exact test, as appropriate. The distribution of etiological categories was also compared with major clinical outcomes, including ICU admission, mechanical ventilation, and in-hospital mortality, using appropriate categorical statistical tests.

Univariable logistic regression analysis was performed to assess the association between potential predictors and in-hospital mortality. Variables considered clinically relevant and/or demonstrating an association with mortality on univariable analysis were subsequently entered into a multivariable logistic regression model to identify independent predictors of in-hospital mortality. Results were reported as odds ratios (ORs) with corresponding 95% confidence intervals (CIs). A two-sided p-value ≤0.05 was considered statistically significant.

## Results

A total of 210 patients with AMS were included, of whom 174 (82.9%) survived to hospital discharge and 36 (17.1%) died during hospitalization. Non-survivors were older and had a greater burden of hypertension, diabetes mellitus, previous stroke, and chronic kidney disease than survivors. No meaningful differences were observed with respect to sex, BMI, duration of AMS, chronic liver disease, or known epilepsy (Table 1).

**Table 1:** Baseline Characteristics of Patients According to In-Hospital Outcome Values are presented as mean±standard deviation (SD) or frequency (%). Continuous variables were compared using the independent-samples t-test, and categorical variables were compared using the Pearson chi-square (χ²) test. Test statistics and two-sided p-values are presented for comparisons between survivors and non-survivors. BMI, body mass index; AMS, altered mental status; SD, standard deviation.

Characteristic	Total (n=210)	Survivors (n=174)	Non-survivors (n=36)	Test statistic	p-Value
Age, years, mean±SD	61.74±16.64	59.60±16.40	72.10±13.80	t=−4.781	<0.001
Male sex, n (%)	118 (56.2)	95 (54.6)	23 (63.9)	χ²=1.046	0.306
Female sex, n (%)	92 (43.8)	79 (45.4)	13 (36.1)	χ²=1.046	0.306
BMI, kg/m², mean±SD	25.56±4.45	25.70±4.40	24.90±4.70	t=0.940	0.352
Duration of AMS, hours, mean±SD	14.81±16.00	13.80±15.20	19.70±18.90	t=−1.759	0.085
Hypertension, n (%)	111 (52.9)	85 (48.9)	26 (72.2)	χ²=6.539	0.011
Diabetes mellitus, n (%)	78 (37.1)	58 (33.3)	20 (55.6)	χ²=6.309	0.012
Previous stroke, n (%)	39 (18.6)	28 (16.1)	11 (30.6)	χ²=4.126	0.042
Chronic kidney disease, n (%)	32 (15.2)	22 (12.6)	10 (27.8)	χ²=5.289	0.021
Chronic liver disease, n (%)	21 (10.0)	15 (8.6)	6 (16.7)	χ²=2.146	0.143
Known epilepsy, n (%)	30 (14.3)	27 (15.5)	3 (8.3)	χ²=1.257	0.262

At ED presentation, non-survivors had poorer neurological status and a less favorable physiological profile than survivors, characterized by lower GCS scores, systolic blood pressure, oxygen saturation, and arterial pH, together with higher heart rate, respiratory rate, body temperature, WBC count, serum creatinine, and serum lactate levels. Random blood glucose and serum sodium did not differ significantly between the two outcome groups (Table 2).

**Table 2:** Clinical and Laboratory Findings at Emergency Department Presentation Values are presented as mean±standard deviation (SD). Continuous variables were compared using the independent-samples t-test. Test statistics and two-sided p-values represent comparisons between survivors and non-survivors. GCS, Glasgow Coma Scale; BP, blood pressure; WBC, white blood cell; SD, standard deviation.

Parameter	Total (n=210)	Survivors (n=174)	Non-survivors (n=36)	Test statistic	p-Value
GCS score	10.79±3.58	11.60±3.10	6.90±3.20	t=8.064	<0.001
Systolic BP, mmHg	126.09±26.45	129.00±25.00	112.00±29.00	t=3.274	0.002
Heart rate, beats/min	94.23±20.48	92.00±19.00	105.00±24.00	t=−3.058	0.004
Respiratory rate, breaths/min	21.34±5.84	20.40±5.10	25.90±7.00	t=−4.475	<0.001
Oxygen saturation, %	93.66±5.57	94.60±4.40	89.10±8.00	t=4.002	<0.001
Body temperature, °C	37.39±1.15	37.30±1.10	37.80±1.30	t=−2.154	0.037
Random blood glucose, mg/dL	161.31±86.88	156.00±83.00	187.00±101.00	t=−1.725	0.091
WBC count, ×10⁹/L	12.07±5.74	11.40±5.20	15.30±7.10	t=−3.127	0.003
Serum sodium, mmol/L	136.75±6.89	137.20±6.50	134.60±8.30	t=1.771	0.084
Serum creatinine, mg/dL	1.52±1.32	1.40±1.20	2.10±1.70	t=−2.352	0.023
Serum lactate, mmol/L	2.73±2.06	2.30±1.60	4.80±2.70	t=−5.364	<0.001
Arterial pH	7.37±0.08	7.38±0.07	7.31±0.12	t=3.383	0.002

Neurological disorders were the most frequent etiological category, followed by infectious and metabolic/endocrine causes. Infectious and cardiopulmonary etiologies were associated with relatively frequent ICU admission, whereas the highest proportion of mortality was observed among patients with multifactorial causes. Overall, 61 (29.0%) patients required ICU admission and 36 (17.1%) died during hospitalization (Table 3).

**Table 3:** Etiological Distribution of Altered Mental Status and Associated Outcomes Values are presented as frequency (%). Percentages for ICU admission and mortality are calculated within each etiological category. The total percentages for ICU admission and mortality represent the proportion of the entire study population experiencing these outcomes. This table is descriptive; no inferential statistical test or p-value was calculated. ICU, intensive care unit; AMS, altered mental status.

Etiological category	Major underlying causes	Patients, n (%)	ICU admission, n (%)	Mortality, n (%)
Neurological	Ischemic stroke, hemorrhagic stroke, seizures/postictal state	61 (29.0)	17 (27.9)	10 (16.4)
Infectious	Sepsis, pneumonia, urinary infection, meningitis/encephalitis	45 (21.4)	18 (40.0)	12 (26.7)
Metabolic/endocrine	Hypoglycemia, electrolyte disturbance, hepatic and uremic encephalopathy	34 (16.2)	7 (20.6)	4 (11.8)
Toxicological/drug-related	Medication toxicity, alcohol, substance intoxication, poisoning	25 (11.9)	4 (16.0)	1 (4.0)
Cardiopulmonary	Hypoxemia, hypercapnia, circulatory compromise	18 (8.6)	8 (44.4)	4 (22.2)
Traumatic	Acute traumatic brain injury	12 (5.7)	3 (25.0)	2 (16.7)
Psychiatric	Acute psychiatric disorders after exclusion of organic pathology	5 (2.4)	0 (0.0)	0 (0.0)
Multifactorial	Two or more contributing etiologies	10 (4.8)	4 (40.0)	3 (30.0)
Total	—	210 (100.0)	61 (29.0)	36 (17.1)

Intravenous fluids and supplemental oxygen were the most frequently used supportive interventions, while antimicrobial and anticonvulsant therapies were also commonly administered. One-fifth of patients required endotracheal intubation and mechanical ventilation, and most patients received specialist consultation. Regarding disposition, approximately half were admitted to the general ward and nearly one-third to the ICU, while fewer than one-fifth were discharged directly from the ED. In-hospital complications occurred in approximately one-fifth of patients, and neurological recovery to or near baseline was documented in nearly two-thirds of the cohort. The mean hospital stay was 6.78±5.42 days (Table 4).

**Table 4:** Emergency Management, Hospital Utilization, and Clinical Outcomes Values are presented as frequency (%) unless otherwise indicated. Length of hospital stay is presented as mean±standard deviation (SD). This table is descriptive; no inferential statistical test or p-value was calculated. ED, emergency department; ICU, intensive care unit; SD, standard deviation.

Management/Outcome	n (%)
Supplemental oxygen therapy	96 (45.7)
Intravenous fluid therapy	142 (67.6)
Empirical or targeted antimicrobial therapy	82 (39.0)
Anticonvulsant therapy	47 (22.4)
Specific antidote administration	18 (8.6)
Vasopressor support	29 (13.8)
Endotracheal intubation/mechanical ventilation	42 (20.0)
Specialist consultation	159 (75.7)
Discharged directly from emergency department	38 (18.1)
Admitted to general ward	111 (52.9)
Admitted to intensive care unit	61 (29.0)
In-hospital complications	46 (21.9)
Neurological recovery to or near baseline	132 (62.9)
Length of hospital stay, days, mean±SD	6.78±5.42
Survived to hospital discharge	174 (82.9)
In-hospital mortality	36 (17.1)

In multivariable logistic regression analysis, poorer neurological status, elevated serum lactate, hypoxemia, hypotension, infectious etiology, and older age remained independently associated with in-hospital mortality. Chronic kidney disease and diabetes mellitus were not independently associated with mortality after adjustment (Table 5).

**Table 5:** Multivariable Logistic Regression Analysis of Independent Factors Associated With In-Hospital Mortality ORs are presented with 95% CIs. Adjusted ORs were obtained from multivariable logistic regression analysis. The Wald chi-square (χ²) statistic was used to assess the significance of independent factors associated with in-hospital mortality in the multivariable logistic regression model. A two-sided p-value ≤0.05 was considered statistically significant. OR, odds ratio; aOR, adjusted odds ratio; CI, confidence interval; GCS, Glasgow Coma Scale; BP, blood pressure.

Predictor	Crude OR (95% CI)	Adjusted OR (95% CI)	Wald χ²	p-Value
Age ≥65 years	2.71 (1.30-5.65)	2.14 (1.02-4.50)	4.037	0.044
GCS score ≤8	6.13 (2.94-12.78)	4.87 (2.19-10.82)	15.091	<0.001
Systolic BP <90 mmHg	3.42 (1.58-7.40)	2.76 (1.18-6.46)	5.480	0.019
Oxygen saturation <90%	4.06 (1.91-8.63)	3.08 (1.34-7.07)	7.030	0.008
Serum lactate >4 mmol/L	4.72 (2.26-9.85)	3.65 (1.62-8.22)	9.765	0.002
Infectious etiology	2.59 (1.22-5.50)	2.32 (1.05-5.13)	4.325	0.038
Chronic kidney disease	2.66 (1.18-5.99)	1.76 (0.71-4.36)	1.491	0.222
Diabetes mellitus	2.50 (1.20-5.22)	1.54 (0.69-3.46)	1.102	0.292

## Discussion

In the present study, AMS was associated with a broad range of neurological, infectious, metabolic, toxicological, cardiopulmonary, traumatic, and psychiatric conditions, with neurological and infectious etiologies accounting for the largest proportions of cases. Overall in-hospital mortality was 17.1%, and 29.0% of patients required ICU admission, highlighting the substantial clinical severity and resource utilization associated with AMS in the emergency setting [16]. Non-survivors were significantly older than survivors (72.10±13.80 vs. 59.60±16.40 years), and age ≥65 years remained independently associated with mortality after multivariable adjustment. Previous stroke, hypertension, diabetes mellitus, and chronic kidney disease were also more frequent among non-survivors. These findings suggest that advanced age and a greater burden of chronic disease may contribute to poorer outcomes in patients presenting with acute alterations in mental status, potentially reflecting reduced physiological reserve and greater susceptibility to complications [16]. Previous studies of older acutely ill patients presenting with acute cognitive dysfunction have similarly reported higher short-term mortality, supporting the prognostic importance of age and comorbidity in this population [17].

Neurological disorders were the most common etiological category in our cohort (29.0%), followed by infectious (21.4%), metabolic/endocrine (16.2%), and toxicological/drug-related causes (11.9%). This distribution emphasizes the heterogeneous nature of AMS and the importance of considering both primary neurological emergencies and systemic causes during the initial emergency assessment. A previous study similarly identified neurological disorders as the leading cause of AMS, accounting for approximately 28% of cases, with toxicological, traumatic, infectious, and metabolic causes also represented [18]. Another study conducted in a comparable clinical setting reported sepsis, cerebrovascular disease, metabolic encephalopathy, and diabetic complications among the principal causes of AMS [19]. Differences in the relative frequency of individual etiologies across studies may reflect variations in patient age, comorbidity profiles, referral patterns, diagnostic resources, and definitions used to classify AMS.

Clinical severity at presentation was strongly associated with in-hospital mortality. Non-survivors had substantially lower GCS scores than survivors (6.90±3.20 vs. 11.60±3.10), together with lower systolic blood pressure and oxygen saturation. They also had higher heart and respiratory rates, WBC counts, serum creatinine, and lactate levels. These findings indicate that patients who died were more likely to present with a combination of severe neurological impairment, hemodynamic compromise, respiratory abnormalities, and systemic physiological disturbance [20]. In multivariable analysis, GCS ≤8 was associated with nearly five-fold higher odds of mortality (aOR 4.87, 95% CI 2.19-10.82). This finding is clinically plausible because a markedly reduced level of consciousness may reflect severe underlying neurological injury or advanced systemic illness and may also increase the need for airway protection and critical care. Previous studies have similarly identified lower initial GCS and markers of greater acute physiological severity as important predictors of adverse outcomes in patients with altered consciousness [21].

Serum lactate was another important prognostic marker in this cohort. Non-survivors had substantially higher lactate concentrations than survivors (4.80±2.70 vs. 2.30±1.60 mmol/L), and lactate >4 mmol/L remained independently associated with mortality (aOR 3.65, 95% CI 1.62-8.22). Elevated lactate may reflect tissue hypoperfusion, impaired oxygen utilization, increased metabolic stress, or the severity of an underlying systemic illness [22]. Its association with mortality in this cohort therefore supports the potential value of lactate as an early marker of physiological severity in patients presenting with AMS. Previous ED data have also demonstrated a graded association between increasing lactate concentration and short-term mortality, with particularly high mortality among patients with lactate concentrations ≥4 mmol/L [22].

Hypoxemia and hypotension were also independently associated with mortality. Patients with oxygen saturation <90% had approximately three-fold higher adjusted odds of death (aOR 3.08, 95% CI 1.34-7.07), while systolic blood pressure <90 mmHg was associated with an aOR of 2.76 (95% CI 1.18-6.46). These associations emphasize the importance of early physiological stabilization in patients with AMS. Hypoxemia and hypotension may represent the effects of severe infection, cardiopulmonary disease, neurological injury, or systemic hypoperfusion and should therefore prompt rapid assessment and treatment of potentially reversible causes [23].

Infectious etiology was independently associated with mortality (aOR 2.32, 95% CI 1.05-5.13). Patients with infectious AMS had a mortality rate of 26.7% and an ICU admission rate of 40.0%. Infection-related AMS may represent an early manifestation of severe systemic infection or sepsis, particularly among older or medically vulnerable patients. The presence of AMS in patients with suspected infection should therefore be considered a potential marker of clinical severity and should prompt timely evaluation for sepsis and other organ dysfunction [24]. The association observed in the present study also reinforces the importance of considering infection even when neurological manifestations predominate at presentation.

The clinical burden of AMS was reflected by the need for substantial acute and inpatient resources. Almost one-third of patients required ICU admission, 20.0% required endotracheal intubation and mechanical ventilation, and 13.8% required vasopressor support. The mean hospital stay was 6.78±5.42 days. Neurological recovery to or near baseline was documented in 132 patients (62.9%), while 82.9% survived to hospital discharge. These findings demonstrate that although most patients survived hospitalization, AMS frequently required intensive monitoring, organ support, and prolonged inpatient care. Previous studies have similarly reported substantial hospitalization requirements and prolonged hospital stays among patients presenting with AMS [25,26].

The present findings have several clinical implications. Initial assessment of patients with AMS should prioritize rapid identification of potentially reversible and life-threatening abnormalities, including hypoglycemia, hypoxemia, hypotension, seizures, infection, and acute neurological pathology. GCS, oxygen saturation, blood pressure, and serum lactate may provide useful early indicators of physiological severity and help identify patients requiring closer monitoring or escalation of care. At the same time, the broad etiological distribution observed in this study supports a systematic diagnostic approach rather than attributing AMS to a single organ system, particularly in older adults with multiple comorbidities.

This study has several limitations. First, its retrospective design required reliance on the completeness and accuracy of existing medical records and therefore introduced the possibility of information and documentation bias. Second, the study was conducted at a single tertiary-care hospital, which may limit the generalizability of the findings to other hospitals or healthcare settings. Third, the sample size was relatively modest, with only 36 in-hospital mortality events. Although multivariable logistic regression was used to adjust for clinically relevant covariates, the relatively small number of outcome events in relation to the number of predictors may have resulted in model overfitting and reduced the precision and stability of the adjusted effect estimates. Therefore, the identified associations should be interpreted cautiously and require validation in larger prospective multicenter cohorts. Fourth, diagnostic investigations were not uniformly performed in all patients; for example, toxicological screening, arterial blood gas analysis, and advanced neuroimaging were performed according to clinical indications. This variation may have resulted in under-recognition or misclassification of some etiologies. Fifth, neurological recovery was assessed during hospitalization rather than through standardized long-term neurological or functional follow-up. Finally, post-discharge mortality and longer-term outcomes were not assessed.

Despite these limitations, this study provides clinically relevant information regarding the etiological spectrum, presenting severity, resource utilization, and short-term outcomes of adults presenting with AMS in a tertiary-care emergency setting. The findings emphasize that neurological and infectious conditions are major contributors to AMS and that severe impairment of consciousness, elevated lactate, hypoxemia, hypotension, infectious etiology, and advanced age are independently associated with in-hospital mortality. Prospective multicenter studies with standardized diagnostic protocols and longer-term follow-up are warranted to validate these prognostic factors and develop reliable risk-stratification tools for patients presenting with AMS.

## Conclusions

Altered mental status was associated with substantial morbidity, intensive care utilization, and in-hospital mortality. Neurological and infectious disorders were the leading etiological categories. Older age, severe impairment of consciousness, hypotension, hypoxemia, elevated serum lactate, and infectious etiology were independently associated with increased odds of in-hospital mortality. A GCS score ≤8 demonstrated the largest adjusted effect estimate among the significant predictors and was strongly associated with mortality. Early recognition of these high-risk features, together with prompt identification and treatment of reversible causes, may facilitate appropriate triage, timely escalation of care, and improved clinical management of adults presenting to the ED with AMS.
